# Cell Non-autonomous Proteostasis Regulation in Aging and Disease

**DOI:** 10.3389/fnins.2022.878296

**Published:** 2022-06-09

**Authors:** Joao Vasco Ferreira, Ana da Rosa Soares, Paulo Pereira

**Affiliations:** Proteostasis and Intercellular Communication Lab, Chronic Diseases Research Centre (CEDOC), NOVA Medical School, Faculdade de Ciencias Medicas, Universidade NOVA de Lisboa, Lisbon, Portugal

**Keywords:** proteostasis, molecular chaperones, transcellular, misfolding, proteotoxicity

## Abstract

Aging is a risk factor for a number of diseases, being the more notorious ones perhaps neurodegenerative diseases such as Alzheimer’s and Parkinson’s. These and other age-related pathologies are often associated with accumulation of proteotoxic material inside cells, as well as with the accumulation of protein deposits extracellularly. It is widely accepted that this accumulation of toxic proteins trails a progressive decline in the mechanisms that regulate protein homeostasis, or proteostasis, during aging. However, despite significant efforts, the progress in terms of novel or improved therapies targeting accumulation of proteotoxic material has been rather limited. For example, clinical trials for new drugs aimed at treating Alzheimer’s disease, by preventing accumulation of toxic proteins, have notoriously failed. On the other hand, it is becoming increasingly apparent that regulation of proteostasis is not a cell autonomous process. In fact, cells rely on complex transcellular networks to maintain tissue and organ homeostasis involving endocrine and paracrine signaling pathways. In this review we will discuss the impact of cell non-autonomous proteostasis mechanisms and their impact in aging and disease. We will focus on how transcellular proteostasis networks can shed new light into stablished paradigms about the aging of organisms.

## Introduction

### Preserving the Proteome

By continuous accumulation of random DNA mutations, selection by survival and reproduction rate, organisms evolved by piecemeal modification of their proteins. In time, proteins increased in variety and complexity, allowing for more intricate enzymatic processes and ever more sophisticated protein assemblies. However, as an extraordinary diverse collection of proteins emerged, organisms struggled to preserve proteome functionality, as higher complexity often compromises structural integrity of proteins. We now know that organisms, from bacteria and archaea to mammals, have developed intricate mechanisms that assist in protein maintenance and quality control, by tightly controlling protein synthesis, supporting the folding of individual proteins and assembly of protein complexes, as well as disposing of damaged or otherwise unwanted ones. This includes, for example, the ribosome-associated quality control factors during synthesis, the aid in folding and support in conformation offered by molecular chaperones or the proteolytic mechanisms that eliminate those proteins rendered obsolete (Balch et al., 2008; Powers et al., 2009; Labbadia and Morimoto, 2015b; Balchin et al., 2016; Deuerling et al., 2019; Jayaraj et al., 2020; Morimoto, 2020; Rebeaud et al., 2021). These and other mechanisms involved in governing protein homeostasis, or proteostasis, comprise an extensive network or around 2000 proteins in humans (Klaips et al., 2018; Hipp et al., 2019). Thus, at the cellular level, a network of proteostasis mechanism is in place to minimize error and maximize efficiency. A malfunction of these mechanisms is extremely detrimental to cellular health and proteostasis loss is often associated with aging and disease.

However, the damage associated to proteostasis breakdown is hardly confined to a group of cells or tissue in an organism. While approaches to the contribution of proteostasis to disease have mostly assumed that regulation of proteostasis networks in living organisms is a cell autonomous process, an overwhelming amount of evidences, gathered over the years, show that organisms organize a proteostasis response a in an integrated, cell non-autonomous level, as well (Taylor et al., 2014; Desdin-Mico and Mittelbrunn, 2017; Takeuchi, 2018; Miles et al., 2019; Miller et al., 2020; Morimoto, 2020). This response involves communication between different cell types, tissues and organs. The advantages of a transcellular proteostasis regulation appear to be manifold. For example, sensorial tissues detect environmental changes that boost proteostasis in sensitive or essential cells for the organism to survive in new and challenging conditions. On the other hand, stress induced proteostasis activation in one tissue can stimulate proteostasis mechanisms in distant tissues, in preparation for stresses to come. Additionally, proteostasis machinery components can be spared from more proteostasis competent cells and delivered to less resilient ones, while cells with failing proteostasis networks can transfer unwanted proteins to neighboring cells and be relieved from the burden (Taylor et al., 2014; Desdin-Mico and Mittelbrunn, 2017; Takeuchi, 2018; Miles et al., 2019; Miller et al., 2020; Morimoto, 2020).

### Protein Synthesis and the Origin of Protein Misfolding

Most proteins need to reach a defined tridimensional structure, or folding conformation, to attain biological function (Hipp et al., 2019). However, the folding kinetics of proteins is constantly being challenged (Morimoto, 2020). While generally the folded (or native) state is thermodynamically favored, often proteins endure challenges that promote additional kinetically stable non-native states (Hipp et al., 2019). Right from the start, biosynthetic errors, which are inherent to protein synthesis, can obstruct protein folding and promote loss of function or toxic gain of function (Morimoto, 2020). Even when successfully translated, specific regions within proteins can be thermodynamically unstable and inherently hard to maintain in the appropriate folding conformation, without assistance from molecular chaperones to maintain conformational stability (Demarest et al., 2002; Dunker et al., 2008; Uversky et al., 2008; Hipp et al., 2019). However, harsh environmental conditions and stresses such as high temperatures and oxidation, as well as additional forms of post-translational modification, are the major drivers for defective folding kinetics. Additionally, a crowded intracellular space promotes non-native interactions and facilitates protein unfolding (Ellis and Minton, 2006; Morimoto, 2020). In humans, cells contain more than 10.000 different proteins with ranging conformational stabilities (Kulak et al., 2017). Moreover, some proteins can reach millions of copies per cell (Ghaemmaghami et al., 2003). Also, individuals within a population contain single-nucleotide variations that may further hinder the folding kinetics of proteins (Lek et al., 2016). Overall, all these different contexts are likely to result in large amounts of metastable polypeptides, with tendency to misfold and create toxic oligomers and aggregates. Therefore, protein conformation and abundance must be strictly controlled, to ensure proper cellular signaling, maintain metabolic flow and the correct assembly of molecular machinery responsible for complex cellular functions such as DNA replication, oxidative phosphorylation and protein synthesis itself.

### Proteome Preservation by Molecular Chaperones

During evolution, as proteins increased in complexity to execute new or more intricate tasks, polypeptide folding efficiency dropped. In fact, the folded conformation of proteins is generally very perilous such that a substantial number of copies of a given protein can exist in partially unfolded states (Hipp et al., 2019). To hold proteome stability and maintain proteins functionally active, molecular chaperones emerged, a family of highly conserved proteins that act by directly interacting with the unstructured polypeptides backbones of proteins in non-native conformations (Hartl, 1996; Rebeaud et al., 2021). The emergence of molecular chaperones supported the structural evolution of proteins into more complex molecules, capable of executing new or improved biological functions. Molecular chaperones can be subdivided into different sub-families (Hartl et al., 2011; Kim Y. E. et al., 2013; Balchin et al., 2016; Carra et al., 2017; Lee et al., 2018). By the direct interaction with client proteins and the hydrolysis of ATP, molecular chaperones improve the folding kinetics of *de novo* synthesized proteins, prevent unfolding and counteract oligomerization/aggregation of polypeptides in non-native conformations.

When under stress, cells can boost the expression of chaperones through specific transcriptions factors such as Heat Shock Factor 1 (HSF1). In steady state, an abundance of inactive chaperones bind to HSF1 to block its activity. Under stress, a surge in chaperone demand titrates chaperones away from HSF1, releasing the transcription factor to command the synthesis of new molecular chaperones (Zou et al., 1998; Anckar and Sistonen, 2011; Zheng et al., 2016; Gomez-Pastor et al., 2018).

Moreover, chaperones are also important in deciding if a client protein has exhausted its time for folding and should be degraded, by coupling molecular chaperones with the ubiquitin-proteasome and autophagy/lysosomal pathways through the ubiquitin ligase CHIP, that binds molecular chaperones and adds ubiquitin chains to client proteins, triggering their degradation (Marques et al., 2006; Ferreira et al., 2013, 2015; Elia et al., 2019; Moran Luengo et al., 2019; Finley and Prado, 2020).

### Elimination of Misfolded Proteins

Elimination of proteins is essential for proteome stability in two major ways, one by adjusting the levels of proteins so they are kept in soluble concentrations (Ciryam et al., 2013, 2015), and a second one by avoiding accumulation of faulty or obsolete proteins (Hipp et al., 2019). Proteins can be degraded by the ubiquitin-proteasome pathway (UPS), whereby proteins tagged with a polyubiquitin chain, conjugated by covalently attaching single ubiquitin moieties by the lysine at position 48, are recognized by the 26S proteasome for degradation (Ciechanover, 1994). Additionally, proteins can be degraded by the autophagic-lysosomal pathway (ALP), a collection of mechanisms that allows for the degradation of individual proteins as well as protein complexes, organelles and even protein aggregates (Ferreira et al., 2013, 2015; Finkbeiner, 2020). Both UPS and ALP directly or indirectly need ATP and use molecular chaperones both to detect unwanted protein species and unfold proteins prior to degradation. Over the years evidences have shown that, in many instances, ALP also uses ubiquitination as a signal for degradation (Kirkin et al., 2009; Ferreira et al., 2013, 2015; Finkbeiner, 2020). While lysine 48 conjugated ubiquitin chains are a signal for proteasomal degradation, lysine 63 and other ubiquitin chains may be a signal for ALP degradation.

### Aging and Proteostasis Decline

Deregulation of the mechanisms governing proteostasis leads to protein malfunction and the formation of toxic protein oligomers and aggregates. There is a strong correlation between proteostasis decline and aging, such that is a feature of many age-related diseases such as Alzheimer’s Disease, Parkinson’s Disease, Age-related Macular Degeneration, Amyotrophic Lateral Sclerosis, and others (Morimoto, 2020). Thus, maintaining proteostasis is essential for organismal health. However, a genetically encoded aging component is evident in *C. elegans* where a sharp change in protein abundance and increase in protein aggregation is clear upon reaching the reproductive age (Walther et al., 2015). Moreover, maintaining proteostasis is energetically costly as well. Both molecular chaperones activity and proteolysis consume ATP. The high energetic cost is consistent with the “disposable soma” theory, whereby organisms might often trade longevity for reproduction, likely by decreasing proteostasis robustness to save energy in favor of progeny generation (Hsin and Kenyon, 1999; Ben-Zvi et al., 2009; Shemesh et al., 2013; Labbadia and Morimoto, 2015a). Additional evidences nonetheless suggest that while there are trade-offs between the soma and the germline, they are not purely based on energy availability (Sala and Morimoto, 2022), as we will discuss later.

In parallel to programmed proteostasis decline, protein aggregation by itself can cause the misfolding of additional proteins in its vicinity, a compound effect exacerbated by post-translation modifications triggered by environmental stress that slowly overburdens proteostasis mechanisms and leads to proteostasis loss (Morimoto, 2020). The combination of programmed decline of proteostasis and environmental stress creates the aging phenotype. The collapse of the proteostasis networks will lead to the accumulation of misfolded, damaged and obsolete proteins, some of which will end up oligomerizing and even aggregating. This phenotype is particularly damaging to post-mitotic cells that cannot use asymmetric cell division to dilute and distribute protein toxicity.

In *C. elegans*, the onset of proteostasis mechanisms failure happens early in life (Ben-Zvi et al., 2009). Chronic expression of misfolded proteins in age-onset neurodegenerative disease leads to accumulation of misfolded species and aggregates that overwhelm proteostasis as a basis of cellular dysfunction (Gidalevitz et al., 2006; Douglas and Dillin, 2010). However, the expression of different aggregation-prone proteins, such as amyloid β or polyQ35 leads to a similar, but not identical, chaperone network activation (Brehme et al., 2014). Surprisingly, chaperone induction upon the expression of aggregative proteins can have opposite effects. For example, torsins 1 and 2 mitigate the toxicity of HD-associated polyQ stretches and the ALS-causing mutated superoxide dismutase 1 (SOD-1), but exacerbate the proteotoxicity of A-beta (Boocholez et al., 2022). This apparent heterogenicity in proteome protection mechanisms reflects the intricacy of age-related diseases and highlights the complexity of developing putative therapeutic approaches.

## Cell Non-autonomous Coordination of Proteostasis

A focus on the study of proteostasis at the cellular level has led to models of proteostasis regulation that skew to cell autonomy. However, in recent years, mounting evidences also indicate that there are mechanisms in place to support a systemic, intercellular and inter tissue, cell non-autonomous variety of networked proteostasis. On one side, pathways regulating systemic proteostasis may control organismal adaptation to stress, by modulating or boosting proteostasis networks in a more even and synchronized manner. On the other, these mechanisms may negatively impact proteostasis by coordinating aging progression and disseminating disease into other cells, tissues and organs. Understanding these mechanisms of systemic proteostasis may offer new and improved ways of boosting proteostasis to mitigate the effects of aging, including the possibility of refining organismal proteostasis by targeting only a small number of cells.

### Systemic Activation of Heat Shock Response

The heat shock response (HSR) is primarily modulated by HSF1, from the heat shock factor (HSF) transcription factor family. HSFs react to increases in temperature, as well as other stresses that can negatively impact protein folding, to recover proteostasis by increasing the amount of available molecular chaperones (Akerfelt et al., 2010).

In a living organism, protection from heat shock also involves changes in behavior, such as moving away from high temperatures. In *C. elegans*, studies in thermolocomotion showed that increasing temperatures activate a pair of amphid finger (AFD) neurons and their postsynaptic amphid Y (AIY) interneurons. In turn, AIY interneurons signal to motor neurons in the muscle wall to promote the movement of organisms away from restrictive temperatures. Surprisingly, inhibition of AFD signaling prevents the HSF-mediated induction of Heat Shock Protein 70 (HSP70) in various non-neuronal tissues of the worm’s body following heat shock, in a process shown to be mediated by serotonin. Additionally, activation of AFD neurons by optogenetics is sufficient to activate HSF1, increase the expression of HSP70 in the whole organism and suppress protein aggregation in muscle tissue (Mori and Ohshima, 1995; Hobert et al., 1997; Inada et al., 2006; Prahlad et al., 2008; Ramot et al., 2008; Prahlad and Morimoto, 2011; Sugi et al., 2011; Tatum et al., 2015).

On the other hand, however, even in the absence of AFD neurons, worms can still activate HSF-1 upon exposure to different stressors, such as the heavy metal cadmium (Prahlad et al., 2008), while AFD incompetent worms were still able to activate the expression of molecular chaperones upon tissue specific expression of polyQ aggregates (Prahlad and Morimoto, 2011). In fact, animals with WT thermosensory neurons express basal levels of chaperones despite the chronic accumulation of misfolded proteins, while retaining their ability to respond to acute heat stress (Prahlad and Morimoto, 2011; Maman et al., 2013). On the other hand mutations in thermosensory neuronal signaling inverts this response, such that chaperone induction in AFD incompetent animals now occurs when misfolded proteins are chronically expressed, but is dampened in response to acute heat stress (Prahlad and Morimoto, 2011; Maman et al., 2013). Therefore, thermosensory neurons are likely to act as neuronal switches for the control of chaperone expression in *C. elegans*, allowing tissues within the organism to maintain optimal levels of chaperones for normal function and yet respond to transient exposures to environmental stress by up-regulating chaperones (Prahlad and Morimoto, 2011; Maman et al., 2013). However, these evidences also highlight that the ability to respond to heat comes at the expense of proteostasis (Prahlad and Morimoto, 2011; Maman et al., 2013). In addition, these observations suggest that systemic proteostasis regulation in *C. elegans* might depend on the particular stress context. Whether this means that different stressors activate different neurons to mediate a proteostasis response, or rather that some stressors preferentially activate cell autonomous mechanisms while others activate cell non-autonomous ones is still a matter of debate.

This might be explained by the fact that additional neurons and neurotransmitters might be implicated in HSR regulation. For example, chemosensory neurons expressing the GPCR thermal receptor 1 (gtr-1) are also involved in systemic HSR activation, as well as inhibiting resistance to stress associated with expression of misfolded proteins (Hobert and Ruvkun, 1998; Beverly et al., 2011; Maman et al., 2013). On the other hand, at the neuromuscular junction, GABA and cholinergic signaling from stimulatory motor neurons can decrease aggregation of polyQ repeats in muscle cells, while the signaling from inhibitory motor neurons acts opposingly to increase polyQ repeats aggregation (Garcia et al., 2007).

Additionally, germline ablation, both in *C. elegans* and drosophila prevents proteostasis collapse in the whole body. This happens because the germline signals to somatic tissues to repress proteome protection, mainly associated to HSF1 inability to bind chaperone promotor regions in the DNA (Flatt et al., 2008; Labbadia and Morimoto, 2015a). However, additional evidences indicate that the impact of reproduction on longevity might be more complex than anticipated (Sala and Morimoto, 2022). For example, the targeted ablation of germ cells leads to lifespan extension, while the abolishment of both germ and somatic gonad cells at the same time fails to do so (Hsin and Kenyon, 1999). In fact, germline ablation acts by activating the longevity gene DAF-19/FOXO in the intestine, through lipophilic hormone signaling (Berman and Kenyon, 2006). In addition, post-translational modifications such as SUMOylation appear to be involved in germline regulation of lifespan (Moll et al., 2018). As far as in vertebrates, a study in zebrafish recently showed that sterile males are more resistant to stress (Chen et al., 2020). Overall, data suggests that there are trade-offs between germline and somatic proteostasis maintenance, even if specific signaling events, rather than solely energetic trade-offs, appear to modulate the longevity of somatic tissues regulated by the germline. In addition, systemic proteostasis regulation appears to involve mechanism of proteome protection as well as, in specific situations, mechanisms of proteostasis depression.

### Systemic Activation of the Endoplasmic Reticulum Unfolded Protein Response

Cell non-autonomous control of proteostasis extends to the UPR*^ER^*. During protein synthesis, proteome surveillance is mainly sustained by the unfolded protein response of the endoplasmic reticulum (UPR*^ER^*). When protein misfolding surpasses a certain threshold, excess misfolded proteins displace the UPR*^ER^* inhibitory chaperone BiP and bind directly to specific membrane receptors: the activating transcription factor 6, the inositol-requiring protein 1, and protein kinase RNA-like ER kinase, leading to the activation of three separate signaling pathways. These conserved pathways have a compound effect that potentiates protein folding such as augmenting ER cisternal space to reduce protein overcrowding, promoting degradation of unwanted proteins, reducing translation and increasing the expression of proteins with the ability to promote and assist protein folding, referred as molecular chaperones (Ron and Walter, 2007; Taylor et al., 2014). However, deletirious mutations of the octamine receptor 1 from *C. elegans* neurons induce increased expression of UPR*^ER^* canonical genes, IRE-1 and X-box Binding Protein 1 (XBP-1), in the whole body (Sun et al., 2012). On the other hand, XBP-1 overexpression in neurons triggers ER chaperone BiP activation both in neurons and the intestine, counteracting the age-related decline of the UPR*^ER^* and promoting longevity (Taylor and Dillin, 2013). Intriguingly, a change in lipid metabolism and an increase in oleic acid is needed to activate systemic UPR*^ER^* when expressing XBP-1 in neurons (Imanikia et al., 2019). In addition, inhibition of neuropeptide secretion by glial cells expressing XBP-1 inhibits UPR*^ER^* activation in distant tissues as well (Frakes et al., 2020). In fact, neuropeptides seem to regulate systemic proteostasis in neurons (Boocholez et al., 2022).

Interestingly, induction of the UPR*^ER^* in non-neuronal tissues is activated in *C. elegans* after pathogen infection, in a mechanism mediated by sensory neurons. In mice, XBP-1 expression in pro-opio-melanocortin neurons activates XBP1 in the liver, resulting in an improved liver function (Williams et al., 2014). In tumor cell lines, UPR*^ER^* activation under stress leads to upregulation of UPR*^ER^* and production of pro-inflammatory cytokines in macrophages, whereas conditioned media from tumor cells induces UPR*^ER^* in dendritic cells, leading to a suppressive phenotype that impairs T cell proliferation and facilitates tumor growth (Mahadevan et al., 2012). The systemic activation of UPR*^ER^* strongly emphasizes the importance of maintaining efficiency of proteins synthesis in a synchronized and cell-non-autonomous fashion in the whole body.

### Systemic Activation of the Unfolded Protein Response of the Mitochondria

Maintaining healthy mitochondria is vital for cell fitness. They are fundamental for energy production, primarily through oxidative phosphorylation. However, the mitochondria electron transport chain is not 100% efficient and leaks electrons that are unable to fully reduce oxygen to form water at cytochrome c oxidase, leading to only partial reduction of oxygen to form the anion superoxide. Thus, mitochondria activity is a major source of reactive oxygen species (ROS). ROS damages proteins and promotes protein misfolding. Accumulation of protein damage impairs mitochondrial activity and overloads to mitochondrial chaperone machinery, to which mitochondria respond by degrading damaged proteins using the mitochondria protease caseinolytic peptidase P (CLPP; Pellegrino et al., 2013). Subsequently, the cleaved peptide products from CLPP action are exported to the cytosol by half transporter 1, that is present at the mitochondrial membrane. Peptide export from mitochondria acts upon the activating transcription factor associated with stress 1, to trigger expression of Unfolded Protein Response of the Mitochondria (UPR*^mito^*) related chaperones (Haynes et al., 2010).

While there are many ways in which the mitochondria ROS can increase *C. elegans* lifespan, including by activating either the hypoxia inducible transcription factor 1 alpha (Lee et al., 2010) or the transcription factor daf-16/FOXO (Senchuk et al., 2018), as well as by lowering ATP production to induce “slow aging” (Yee et al., 2014), lifespan can be extended in a systemic fashion, through cell-non-autonomous regulation of UPR*^mito^*. Depletion from neurons of the cco-1 protein, a component of the mitochondria electron transport chain, is sufficient to activate UPR*^mito^* in the gut, as well as increased lifespan (Durieux et al., 2011). Depletion of cco-1 in the whole organism leads to the same increase in the lifespan in the worm (Durieux et al., 2011). Interestingly, depletion of cco-1 from body wall cells has the opposite effect, reducing lifespan (Durieux et al., 2011). This observation indicates that similar stresses applied to different areas of the body do not wield the same proteome protective measures, and that efficiency of systemic proteostasis activation might depend on both the type of stress and the tissue that is most affected by stress.

On the other hand, expression of Huntington causing protein in polyQ 40-repeat protein in neurons activates the expression of mitochondrial HSP70 (mtHSP70) in the gut of *C. elegans*, in a process mediated by serotonin (Berendzen et al., 2016). Other groups found additional factors that mediate systemic UPR*^mito^* activation in *C. elegans*, such as Wnt signaling and neuropeptide FLP-2 (Shao et al., 2016; Zhang et al., 2018).

In mammals, fibroblast growth factor 21 is responsible for signaling to peripheral tissues upon mitochondrial damage in muscle cells, leading to resistance to obesity and improved insulin sensitivity, which might indicate a systemic activation of UPR*^mito^* (Kim K. H. et al., 2013).

### Transcellular Chaperone Signaling

Other forms of cell non-autonomous proteostasis regulation involve molecular chaperone signaling that is unrelated to HSF-1 activation. For example, in *C. elegans* specific over-expression of HSP90 in the gut or in neurons protects against misfolding of myosin and amyloid β aggregation in the muscles of the body wall. In neurons, for example, this happens because HSP90 overexpression leads to PQM-1 activation (zinc finger transcription factor) and subsequent HSP90 upregulation in distant tissues by glutamatergic signaling (Taylor and Dillin, 2013; van Oosten-Hawle et al., 2013; O’Brien et al., 2018).

A second form of transcellular chaperone signaling is the direct secretion of molecular chaperones by cells. Initial evidence in mice showed that overexpression of HSP40 in one brain region of polyQ diseased animals lead to the elimination of inclusion bodies in other parts of the brain (Popiel et al., 2012). In drosophila, expression of HSP70 and HSP40 in muscles and fat cells prevented eye degeneration caused by polyQ proteins (Warrick et al., 1999; Kazemi-Esfarjani and Benzer, 2000). These initial observations eventually lead to the discovery that molecular chaperones are secreted to the extracellular space, encased inside a subtype of extracellular vesicles called exosomes (Takeuchi et al., 2015). Exosomes are small extracellular vesicles that are present in virtually all biological fluids and circulate in the blood stream. However, not all chaperones are secreted *via* this non-canonical secretory pathway, only cytosolic ones. Exosomes loaded with cytosolic chaperones are able to get endocytosed by other cells and suppress aggregate formation by polyQ proteins. In drosophila, inhibition of exosome secretion from muscle and fat cells expressing HSP70 and HSP40 suppressed eye regeneration (Takeuchi et al., 2015).

The molecular basis for the loading and secretion of chaperones *via* exosomes is still unclear. However, reports show that depletion of HSC70 from cells decreases HSP40 presence in exosomes (Kampinga and Craig, 2010). Additionally, a recent paper demonstrates that HSC70, and potentially other chaperones that directly interact with HSC70 (such as HSP40, HSP90, and others), are loaded into nascent exosomes by a mechanism dependent on the endo-lysosomal transmembrane receptor LAMP2A (Ferreira et al., 2022). These observations indicate that there is a conserved molecular mechanism for the loading and transfer of molecular chaperones *via* exosomes, with the potential to boost proteostasis in a cell non-autonomous way.

### Transcellular Transfer of Proteotoxic Material

The secretion of misfolded, oligomerized, and/or aggregated material to the extracellular space by vesicles, particularly by exosomes, has been proposed as a new major pathway for the disposal of unwanted proteins. Overburden cells with falling proteostasis capabilities would secrete unwanted and toxic protein material encased inside exosomes. Exosomes originate from multivesicular endosomal compartments and are therefore at the crossroads between protein degradation by the lysosome and vesicle secretion. Analysis of the protein cargo of exosomes supports this hypothesis. Misfolded and prion proteins (PrP) are released in exosomes (Guo et al., 2015), particularly the ones implicated in neurodegenerative diseases such as Huntington’s, Alzheimer’s and Parkinson’s disease, including amyloid β, APP C-terminal fragments, Tau, α-synuclein, SOD1, and the PrP (Fevrier et al., 2004; Rajendran et al., 2006; Perez-Gonzalez et al., 2012; Saman et al., 2012; Grad et al., 2014). Additionally, oligomerized proteins have been shown to be secreted by exosomes upon ubiquitin ligase CHIP inactivation (Ferreira et al., 2019). While the initial effect of the secretion of these proteins might be beneficial, particularly for post-mitotic cells such as neurons and the retina pigmented epithelium, in some instances and over time, such mechanisms could do more harm than good, participating in the spreading of disease rather than in the dilution of the proteotoxic burden.

The mechanisms that regulate the loading of proteotoxic material into nascent exosomes are still a matter of debate. One way this could happen is through the trapping of misfolded proteins bound to the molecular chaperone HSC70, that is included in exosomes, either through the interaction with LAMP2A or by attaching to lipids, in the endosomal limiting membrane, where exosomes are formed (Sahu et al., 2011; Ferreira et al., 2022).

## Concluding Remarks

**FIGURE 1:**
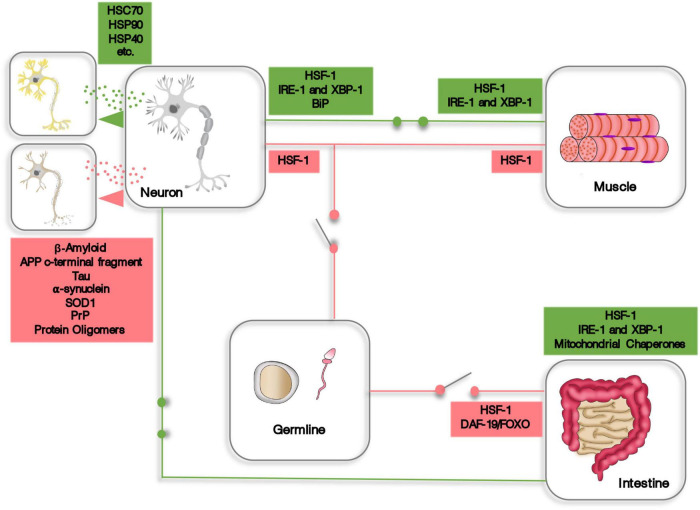
A schematic representation of some aspects of cell non-autonomous proteostasis. Expression of HSF-1, XBP-1, and other components of the proteostasis machinery is induced (green circuits) or repressed (red circuits) in one tissue or organ by endocrine signaling originating from distant cells. In addition, cells and tissues can transfer proteostasis machinery or dispose of proteotoxic material using extracellular vesicles.

While there is still much to be discovered regarding cell non-autonomous proteostasis, there are definitely an overwhelming amount of evidences showing that organisms can build up from cellular based proteostasis mechanisms to a fully integrated and systemic proteostasis network, encompassing the whole body. These transcellular coordinated responses involve endocrine and paracrine intercellular and intertissue communication, either through signaling molecules or by the material exchange of proteostasis machinery and proteotoxic peptides *via* extracellular vesicles (Figure 1). Intriguingly, integrated proteostasis networks can function as synchronized proteome protector mechanisms and, opposingly, coordinate proteostasis decline, such as in the example of the germline signaling to inhibit HSF1 activity. These evidences highlight the complexity and intricacy of organismal proteostasis and how important the study of cell non-autonomous proteostasis is. Therefore, we do believe that future research aimed at mitigating the effects of aging should focus on the systemic aspects of proteostasis to generate new options for innovative and more targeted therapeutic strategies.

## Author Contributions

JF conceptualized and wrote and proofread the manuscript. AR and PP conceptualized and proofread the manuscript. All authors contributed to the article and approved the submitted version.

## Conflict of Interest

The authors declare that the research was conducted in the absence of any commercial or financial relationships that could be construed as a potential conflict of interest.

## Publisher’s Note

All claims expressed in this article are solely those of the authors and do not necessarily represent those of their affiliated organizations, or those of the publisher, the editors and the reviewers. Any product that may be evaluated in this article, or claim that may be made by its manufacturer, is not guaranteed or endorsed by the publisher.
